# Invasive Cryptococcosis and Adalimumab Treatment

**DOI:** 10.3201/eid1306.070154

**Published:** 2007-06

**Authors:** Juan P. Horcajada, Jose L. Peña, Víctor M. Martínez-Taboada, Trinitario Pina, Isabel Belaustegui, María Eliecer Cano, Daniel García-Palomo, M. Carmen Fariñas

**Affiliations:** * University Hospital Marqués de Valdecilla, Santander, Spain

**Keywords:** Cryptococcosis, adalimumab, rheumatoid arthritis, letter

**To the Editor:** Tumor necrosis factor-α (TNF-α) antagonists are immunosuppressants that have shown efficacy in treating inflammatory disorders. However, a recent meta-analysis of controlled trials has shown evidence of increased risk for serious infections in patients with rheumatoid arthritis treated with TNF-α antagonists (*1*).

Adalimumab is a human monoclonal antibody to TNF-α approved by the US Food and Drug Administration (FDA) for treatment of rheumatoid arthritis. The Spanish registry of adverse events of biologic therapies in rheumatic diseases reported that 1,080 patients were treated with adalimumab from 2003 through 2006 and no cases of cryptococcosis were recorded (*2*). No cases of cryptococcosis have been detected in 10,050 treated patients in the US postmarketing database for adalimumab (*3*). We report invasive cryptococcosis in a patient receiving adalimumab. This case underscores the relationship between TNF antagonists and emergence of severe and difficult-to-treat opportunistic infections.

A 69-year-old woman with rheumatoid arthritis diagnosed in 2002 was referred to our hospital for severe acute inflammation of the second finger of the left hand. She had been treated with oral corticosteroids (prednisone, 7.5 mg/day) and several disease-modifying antirheumatic drugs, including chloroquine, methotrexate, and sulfasalazin, without improvement. One year before the current episode, therapy with adalimumab, 40 mg subcutaneously every 2 weeks for 52 weeks, was started and she showed an acceptable clinical response. She had no recent trauma.

Examination showed severe tenosynovitis of the digital flexor tendon with intense edema and compartmental signs (Appendix Figure). She had an axillary temperature of 36.7°C and an admission leukocyte count of 5,900 cells/µL. Results of a neurologic examination and a chest radiograph were normal. Early surgical decompression was performed. Intraoperative findings indicated extensive subcutaneous celullitis with infiltration of vasculonervous bundles and flexor tendon synovitis. Culture of extracted material from 4 samples, including a biopsy specimen of subcutaneous tissue, identified *Cryptococcus neoformans* susceptible to amphotericin B, azoles, and flucytosine. Results of cerebrospinal fluid analysis were normal. A cranial computed tomographic scan showed no focal lesions. Results of a serum cryptococcal latex test and HIV serologic analysis were negative. Magnetic resonance imaging of the finger showed inflammation of soft tissues, including the flexor tendon, but no signs of arthritis or osteomyelitis. Treatment with adalimumab was discontinued.

Intravenous liposomal amphotericin B, 300 mg once a day, and intravenous flucytosine, 2.5 g 3× a day, were administered for 7 days. Treatment with intravenous fluconazole, 400 mg twice a day for 21 days, was then started. Inflammatory signs decreased. Because residual soft tissue necrosis was extensive, reconstructive surgery was not performed, and her second finger was amputated during the third week after admission. A pathologic examination showed chronic necrotizing granulomatous inflammation with typical encapsulated fungal forms of *Cryptococcus* spp. inside multinucleated giant cells. These forms were observed by staining specimens with hematoxylin and eosin and Mayer mucicarmine (Figure). After an uneventful postoperative period, the patient was discharged and received oral fluconazole, 200 mg once a day for 6 months. Two years later, the patient remains asymptomatic and receives therapy with methotrexate, salazopyrin, and prednisone.

**Figure:**
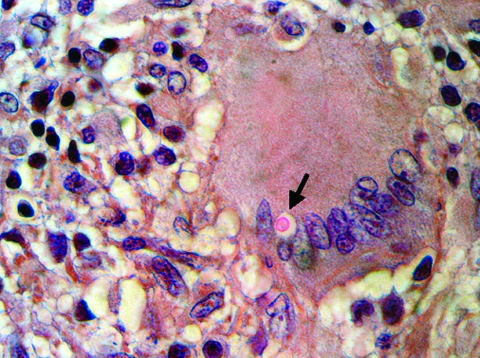
Histiocytic granuloma with lymphocytes and multinucleated giant cells and an encapsulated intracytoplasmic mucicarmine-positive structure identified as a *Cryptococcus* sp. (arrow) (hematoxylin and eosin– and Mayer mucicarmine–stained, magnification 400×).

The rate of serious infections in the US clinical trial safety database of adalimumab as of April 2005 was 5.1/100 patient-years. This rate is similar to that reported in the general population with rheumatoid arthritis. However, as in our case, some infections associated with adalimumab are severe and difficult to treat (*3*). Cryptococcosis has not been previously associated with use of adalimumab. Cryptococcal infections have been described in 19 patients receiving TNF-α antagonists other than adalimumab (infliximab or etanercept) in the FDA Adverse Event Reporting System from 1998 to 2002 (*4*). Three cases of cryptococcosis in patients receiving TNF-α antagonists have been reported (*5*–*7*).

The association between cryptococcosis and use of TFN-α antagonists can be explained by the immune response to *C*. *neoformans*, which relies on effective T-cell host defenses and in which TNF-α has an essential role. TFN-α is involved in maintaining a T-helper cell type 1 immune response because it induces production of interleukin-12 (IL-12) and IL-18, with subsequent production of fungicidal interferon-γ (*8*). In animal models, TNF-α blockers are associated with reduced recruitment of inflammatory cells to the area of infection and an increased risk for cryptococcal dissemination (*9*). Moreover, *C*. *neoformans* impairs production of TNF-α, IL-1β, and IL-6 and increases levels of IL-10, which induce a T-helper cell type 2 immune response (*10*). Cryptococcal virulence factors impart greater dependence upon TNF-α for a sufficient host response (*9*). Adalimumab may increase immunosuppression, which is required for a cryptococcal infection.

Our patient received a low dose of prednisone. Although corticosteroids are a risk factor for cutaneous cryptococcosis, cases with serious outcomes rarely occur. However, the risk for fungal infection related to low doses of steroids is minimal. Active surveillance, as well as analysis of associated risk factors, is required to detect concurrence of severe opportunistic infections in patients treated with TNF antagonists and to identify patients who could benefit from these therapies with fewer risks.

## Supplementary Material

Appendix Figure Severe acute tenosynovitis of the flexor tendon of the second finger of the left hand of the patient.
