# Supplementary figures and images for: Invasive Cryptococcosis and Adalimumab Treatment
Source: Emerg Infect Dis. 2007 Jun;13(6):953–5. doi: 10.3201/eid1306.070154 (PMC2792838; doi:10.3201/eid1306.070154)

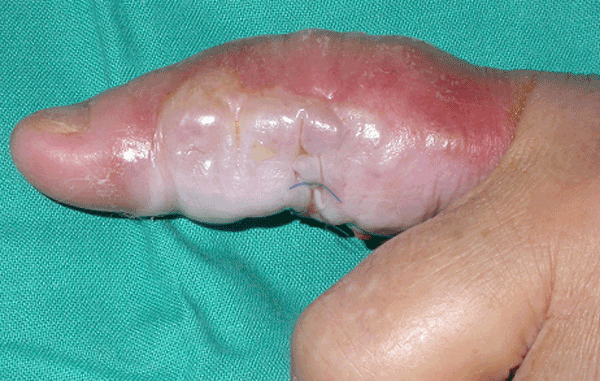

Supplement: Appendix Figure — Severe acute tenosynovitis of the flexor tendon of the second finger of the left hand of the patient. [file 07-0154_appF-s1.gif]
